# Visual Global Processing and Subsequent Verbal and Non-Verbal Development: An EEG Study of Infants at Elevated versus Low Likelihood for Autism Spectrum Disorder

**DOI:** 10.1007/s10803-022-05470-w

**Published:** 2022-03-30

**Authors:** Martina Hedenius, Irzam Hardiansyah, Terje Falck-Ytter

**Affiliations:** 1 Department of Public Health and Caring Sciences, Speech-Language Pathology, Uppsala University, P.O. Box 564, 752 37 Uppsala, Sweden; 2 Karolinska Institutet Center of Neurodevelopmental Disorders (KIND), Centre for Psychiatry Research, Department of Women’s and Children’s Health, Karolinska Institutet, & Stockholm Health Care Services, Stockholm County Council, CAP Research Centre, Gävlegatan 22, 113 30 Stockholm, Sweden; 3 Development and Neurodiversity Lab (DIVE), Department of Psychology, Uppsala University, Uppsala, Sweden; 4 The Swedish Collegium for Advanced Study (SCAS), Uppsala, Sweden

## Introduction

Global Motion Processing (GMP) refers to the combination of local visual motion signals into a global, coherent perception of motion (Goodale & Milner, 1992). This perceptual ability emerges gradually early in infancy, and is thought to be involved in a range of developmentally important higher-order skills such as the visual guidance of movement (e.g., as when trying to grasp an object) and the perceptual integration of facial movement (Shah et al., 2016). Sensitivity to visual motion signals has been demonstrated in infants as young as 2 months of age (Blumenthal et al., 2013; Braddick et al., 2003). However, adult-like levels of processing may not be established until late childhood (Armstrong et al., 2009; Hadad et al., 2011).

Global Motion Processing is commonly assumed to provide an index of the function of the visual dorsal stream, and is as such often contrasted with Global Form Processing (GFP), thought to rely on the visual ventral stream (Mishkin & Ungerleider, 1982; Goodale & Milner, 1992). Although the two visual streams are anatomically, as well as functionally, interconnected (Cloutman, 2013; van Polanen & Davare, 2015), they are generally considered to underlie at least partly distinct visual functions. The dorsal stream is typically associated with motion perception and visuomotor integration, and includes area V1, motion-sensitive area V5, and areas within the posterior parietal lobe (Hadad et al., 2015). The ventral stream, in contrast, has been shown to support form processing and object recognition (Mishkin & Ungerleider, 1982; Goodale & Milner, 1992), and involves area V1 projections to the inferior temporal lobe.

An association between GMP and motor and language development may be theoretically plausible for several reasons. With respect to motor development, the dorsal stream has long been implicated in the processing and execution of movement (e.g., Goodale & Milner, 1992; Hadad et al., 2015). In line with this, several studies have found concurrent associations between GMP and motor skills in children with typical as well as atypical development. For example, Chakraborty et al. (2017) examined GMP and motor skills in 4.5-year old children exposed to a variety of environmental risk factors for atypical development. The GMP paradigm was a standard random dots kinematograms paradigm in which the children observed dots on a screen, some of which moved in a coherent direction, and some that moved randomly. On each trial, the children were asked to judge the direction of the moving dots. The proportion of coherently moving dots in each trial varied systematically in order to decide the motion coherence threshold (i.e., the lowest ratio required for detection of coherent motion) for each child. The authors found a positive association between motion coherence thresholds and the children’s scores on standardized tests of visuo-motor integration, motor coordination, and gross motor skills. This finding was replicated by Thompson et al. (2017) with a similar study design in 2-year old children with typical development. Although these studies did not directly test an association between GMP and language development, analyses including verbal IQ as a covariate indicated that GMP was also associated with children’s language abilities.

There are several potential pathways that could underlie an association between GMP and language development. For example, the effects of GMP difficulties on the perceptual integration of facial movement may lead to downstream effects on socio-cognitive development (Shah et al., 2016), which, in turn, is closely linked to early language development (Taylor et al., 2020). Additionally, difficulties with facial movement processing could impact language development via the ability for visual speech-processing (Borowiak et al., 2018). Moreover, evidence suggests that GMP relies on other, possibly domain-general, capacities that may also be critical for early language development. Such capacities include attention (Maunsell & Treue, 2006), the integration of information over space and time (Happé & Frith, 2006), signal-from-noise segregation and predictive coding (Van de Cruys et al., 2017).

The dorsal stream vulnerability hypothesis (Braddick et al., 2003) proposes that the dorsal stream is relatively more susceptible to the effects of atypical neurodevelopment compared to the ventral stream. Global motion processing atypicalities are apparent in neurodevelopmental conditions such as William’s Syndrome (Atkinson, 2017; Atkinson et al., 1997), developmental dyslexia (Benassi et al., 2010) and autism spectrum disorder (ASD; Spencer et al., 2000; for a review see Van der Hallen et al., 2019), while abnormalities in GFP for these populations are rarely reported. Despite their heterogeneity, these disorders share characteristic difficulties in motor and language function (Karmiloff-Smith et al., 2003; Tager-Flusberg & Caronna, 2007; Torppa et al., 2010; Wilson et al., 2018), which may relate to GMP.

Autism spectrum disorder is an early-onset neurodevelopmental condition that is characterized by difficulties with social reciprocity and social communication, and rigid, repetitive patterns of behavior, interests or activities (DSM-5; American Psychiatric Association, 2013). Such communication-based and repetitive motor symptomatology could potentially be related to deficits in GMP. However, previous research on GMP in ASD has yielded inconsistent results, with some studies showing reduced performance in the ASD group compared to a control group (e.g., Milne et al., 2002; Pellicano et al., 2005; Spencer et al., 2000), and other studies finding no group differences (e.g., Brieber et al., 2010; Jones et al., 2011). In a recent meta-analysis of studies focusing on global and biological motion in ASD, Van der Hallen et al. (2019) found ASD to be associated with a deficit of a small effect size that was not moderated by the type of paradigm (i.e., global motion or biological motion). Several studies have shown considerable individual variability in the ASD group, suggesting that some of this inconsistency could be explained by differences in sample characteristics between studies. In line with this, there is evidence to suggest that problems with GMP in ASD may be linked to verbal and non-verbal IQ (Koldewyn et al., 2010) as well as to a history of early language delay (Takarae et al., 2008). Somewhat surprisingly, however, neither verbal IQ nor any of the other moderators relating to task and participant characteristics (e.g., type of task, performance IQ, and age) reached significance in the meta-analysis by Van der Hallen et al., (2019), and further research is needed to clarify the sources of GMP variability within the ASD population. The status of GFP in ASD is also unclear. Although there are some indications that GFP may also be affected in ASD (e.g., Spencer & O'Brien, 2006), other studies have found intact GFP in the condition (e.g., Koldewyn et al., 2010; Jones et al., 2011).

Recently, Nyström et al. (2021) used electroencephalogram (EEG) to map the topographical distribution of electrical activity related to GMP and GFP in 5-month-old infants with elevated likelihood (EL) of ASD (i.e., with an older sibling diagnosed with ASD; Ozonoff et al., 2011) and infants at low likelihood (LL; i.e., with no first- or second-degree relative with ASD). Activity was recorded from the primary and extrastriate areas that have been shown to be involved in the integration of output from the primary visual cortex (Wattam-Bell et al., 2010) while infants watched a screen on which GMP and GFP stimuli alternated with local motion/form stimuli. The stimuli consisted of 2000 local arcs, each of which was made up of eight white dots plotted on a dark background. When the dots were plotted simultaneously on the screen, they formed a global concentric texture, which constituted the GFP condition. The GMP condition was created when the dots were plotted successively, forming a globally rotating motion around the center of the screen (Nyström et al., 2021; Wattam-Bell et al., 2010). The results revealed a more lateralized brain activation pattern (i.e., with a more left- or right-topographical distribution) in the EL group, compared to the LL group, in response to both the GMP and GFP conditions. Notably, there is evidence to suggest that atypical lateralization of motor function in ASD is linked to atypical performance at the behavioral level (Floris et al, 2016). This suggests that the atypical lateralization of electrical activity in the EL group that was observed by Nyström et al. (2021) could be associated with processing atypicalities at the functional level.

In the current study, we examined whether variability in the topographical organization of GMP/GFP at 5 months (Nyström et al., 2021) is associated with children’s motor and language abilities at 18 months. Based on the research outlined above which suggests links between GMP and motor as well as language skills, and between atypical lateralization and brain function, we hypothesized that a high degree of atypical GMP lateralization at 5 months would be associated with lower motor as well as language scores at 18 months, whereas no such associations would be found for GFP. In an exploratory arm of the study, the relationship between GMP/GFP and visual cognition was examined. For these exploratory analyses, we formed no a priori hypothesis regarding the outcome.

## Methods

### Ethical Considerations

All parents gave written informed consent before participation. The study was performed in accordance with the Declaration of Helsinki and was approved by the Ethics Board in Stockholm.

### Participants

**Fig. 1:**
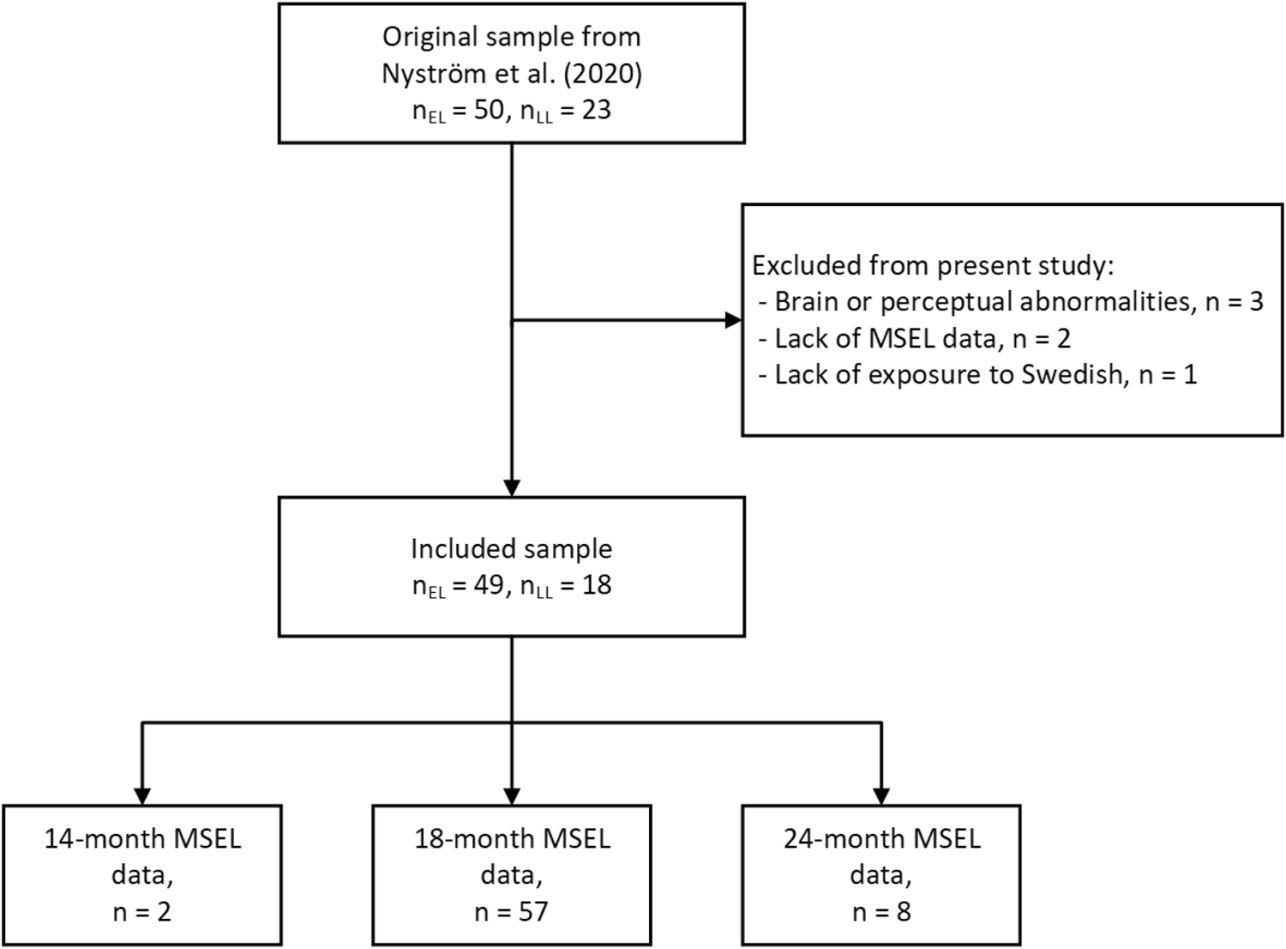
Flow chart of the participant recruitment procedure. *EL* = Children at elevated likelihood of autism; *LL* = Children at low likelihood of autism; *MSEL* = Mullen Scales of Early Learning (Mullen, 1995). To increase the number of children included in the follow up analyses, we included MSEL from 14 or 24 months if 18 m data was not available (see Methods)

As previously described in Nyström et al. (2021), the participating families were recruited within the Early Autism Sweden (EASE) project (Falck-Ytter et al., 2018). The original sample included in the Nyström et al. (2021) study were 50 infants with elevated likelihood (EL), and 23 infants with low likelihood (LL) for ASD (Fig. 1). All infants in the EL group had at least one older sibling diagnosed with autism, while those in the LL group had no first- or second-degree relatives with such a diagnosis. For the present study, six children were excluded due to (i) documented brain or perceptual abnormalities (periventricular leukomalacia: *n* = 1; hearing loss: *n* = 1; visual impairment: *n* = 1), (ii) lack of data from the Mullen Scales of Early Learning (MSEL; Mullen, 1995; *n* = 2), and (iii) lack of exposure to the Swedish language (*n* = 1). Thus, the final sample in the present study included 67 infants, with 49 participating in the EL group, and 18 in the LL group. For 10 of the included children, 18 months MSEL data were missing. In these cases, we used MSEL data from the latest available time point. For eight children (El *n* = 7; LL *n* = 1), the latest time point was 24 months, and for two LL children it was 14 months. Table 1 displays participant characteristics for the final sample of children included in the analyses.

**Table 1:** Participant characteristics

Variable	EL (*n* = 49)		LL (*n* = 18)		Comparison	
	*M*	*SD*	*M*	*SD*	*t*	*p*
Sex (F/M)	27/22		9/9		*χ*^2^ = 0.14	0.710
MSEL_Age^a^	585	80	549	65	1.80	0.075
MSEL_GM^b^	51.0	5.7	50.4	9.9	0.34	0.738
MSEL_FM^c^	53.7	7.5	53.4	7.8	0.15	0.877
MSEL_RL^d^	46.1	14.1	49.9	12.9	1.01	0.313
MSEL_EL^e^	43.8	9.2	45.3	8.5	0.64	0.527
MSEL_VR^g^	49.9	8.1	51.2	9.9	0.57	0.572
GMP_LAT^h^	0.23	0.17	0.14	0.15	2.06	0.043
GFP_LAT^i^	0.24	0.17	0.10	0.20	2.89	0.005

^a^Age at MSEL assessment in days

^b^MSEL Gross motor subscale score

^c^MSEL Fine motor subscale score

^d^MSEL Receptive language subscale score

^e^MSEL Expressive language subscale score

^g^MSEL Visual reception subscale score

^h^Global motion processing laterality score

^i^Global form processing laterality score

### Stimuli and Procedure

#### Topographical Organization of GMP at 5 Months

A detailed description of the stimuli and procedure for the assessment of the topographical organization of GMP and GFP can be found in Nyström et al. (2021). In brief, the EEG was recorded using an age appropriate 128-channel Geodesic Sensor Net (HCGSN 130; EGI, Eugene, OR). Stimuli were generated by a MacBook Pro using the PsychToolbox in MATLAB (2013a), and presented on a BenQ (23.5 inches) monitor with 1920 × 1080 pixel resolution operating at 60 Hz frame rate. For both form and motion, 2000 local arcs were always present on the screen, alternating between coherent motion/form and random coherent displacement every 250 ms. Each local arc consisted of eight white dots plotted on a dark background (0.29° visual degrees). Following an 8-frame lifetime, each dot was replotted in a fresh random location on the screen. When plotted simultaneously on the screen, these dots created a short, static arc segment (the GFP condition). When plotted successively, they created a brief sample of motion along an arc trajectory for the GMP condition (displacement between frames gave a speed of 8.6 visual degrees/s). In the GFP condition the coherent interval resulted in a global concentric texture, and in the GMP condition the coherent interval created a globally rotating motion about a common origin at the center of the screen. Patterns were viewed at ~ 60 cm and subtended 47.4° × 27.8°. The stimuli were presented in 12-s blocks, containing 24 cycles. Each 12-s block contained only GFP or GMP stimuli, to entrain neural responses to the frequency of the specific condition, and the blocks were interleaved with unrelated experimental stimuli. Ten GFP blocks and 10 GMP blocks were presented, giving 240 cycles in total for each condition.

Brain responses related to the stimuli were extracted by calculating the T2 statistics (Victor & Mast, 1991) for all cycles and channels separately. The assessment of the topographical distribution of brain responses was based on T2circ values by interpolating the electrode values over a uniform grid (~ 3500 vertices), bound by the outer electrodes of the nets. These interpolated values were averaged separately within nine areas of interest (AOIs) around the back of the head. Each participant’s data consisted of separate 9-point spatial brain activity profiles that were vector-normalized (McCarthy & Wood, 1985) to eliminate overall amplitude differences between individual infants. The measure of topographical distribution of the GFP and GMP signals was calculated by subtracting the T2 circ value of the central AOI from the maximum T2circ across all other AOI (i.e. AOIs 1–4 and 6–9).

#### Assessment of Motor, Language, and Visual Abilities at 18 Months

The children’s motor, language, and visual cognition outcomes at 18 months were assessed with the Mullen Scales of Early Learning (MSEL; Mullen, 1995). This test measures aspects of nonverbal and verbal development in children from 0—68 months of age in five domains: gross motor, fine motor, receptive language, expressive language, and visual reception. Standard scores (T-scores) with a mean of 50 and standard deviation of 10 were used in the analyses (Mullen, 1995). A motor composite score was calculated for each participant based on the mean of the gross and fine motor subscale T-scores. Similarly, a language composite score was obtained based on the mean of the receptive and expressive language subscale T-scores.

## Statistical Analyses

The hypothesis of an association between the degree of early atypical lateralization of GMP, and later motor and language scores, was tested with two independent linear regression models. In both models, the independent variables were the degree of GMP and GFP laterality, operationalized as the normalized T2circ value of AOI5 subtracted from the max T2circ value across the remaining AOIs (see Sect. 2.3.1). The independent variables were not correlated (*r* = 0.042, *p* = 0.736). In the first model, the effect on motor ability was tested by using the mean T-score of MSEL gross and fine motor subscales as the dependent variable. In the second model, the dependent variable was the mean T-score of the expressive and receptive language subscales. Each analysis was repeated with group and sex added as categorical covariates, allowed to interact with GMP and GFP, to explore potential moderating effects of these variables on the models. The significance level for these hypothesis-driven analyses was set to *p* < 0.05.

Finally, in order to explore potential predictive effects of GMP/GFP laterality on visual cognition, the analyses described above were repeated using MSEL visual reception T-scores as the dependent variable. The significance level for this exploratory analysis was set to *p* = 0.025.

## Results

### The Predictive Relationship Between GMP/GFP and Motor Ability

The regression analysis with GMP and GFP laterality as continuous independent variables and the mean T-score of the MSEL gross and fine motor subscales as the dependent variable produced no significant effects (main effect of GMP laterality: *F*[1, 64] = 0.052, *p* = 0.820, $\eta_\rho^2$ = 0.0008; main effect of GFP laterality: *F*[1,64] = 0.370, *p* = 0.545, $\eta_\rho^2$ = 0.006). Adding group and sex as covariates in the model did not significantly affect this pattern (main effect of GMP laterality: *F*[1, 56] = 0.002, *p* = 0.960; main effect of GFP laterality: *F*[1,56] = 3.60, *p* = 0.063. All other main effects and interactions: *p*s > 0.05).

### The Predictive Relationship Between GMP/GFP and Language Ability

**Fig. 2:**
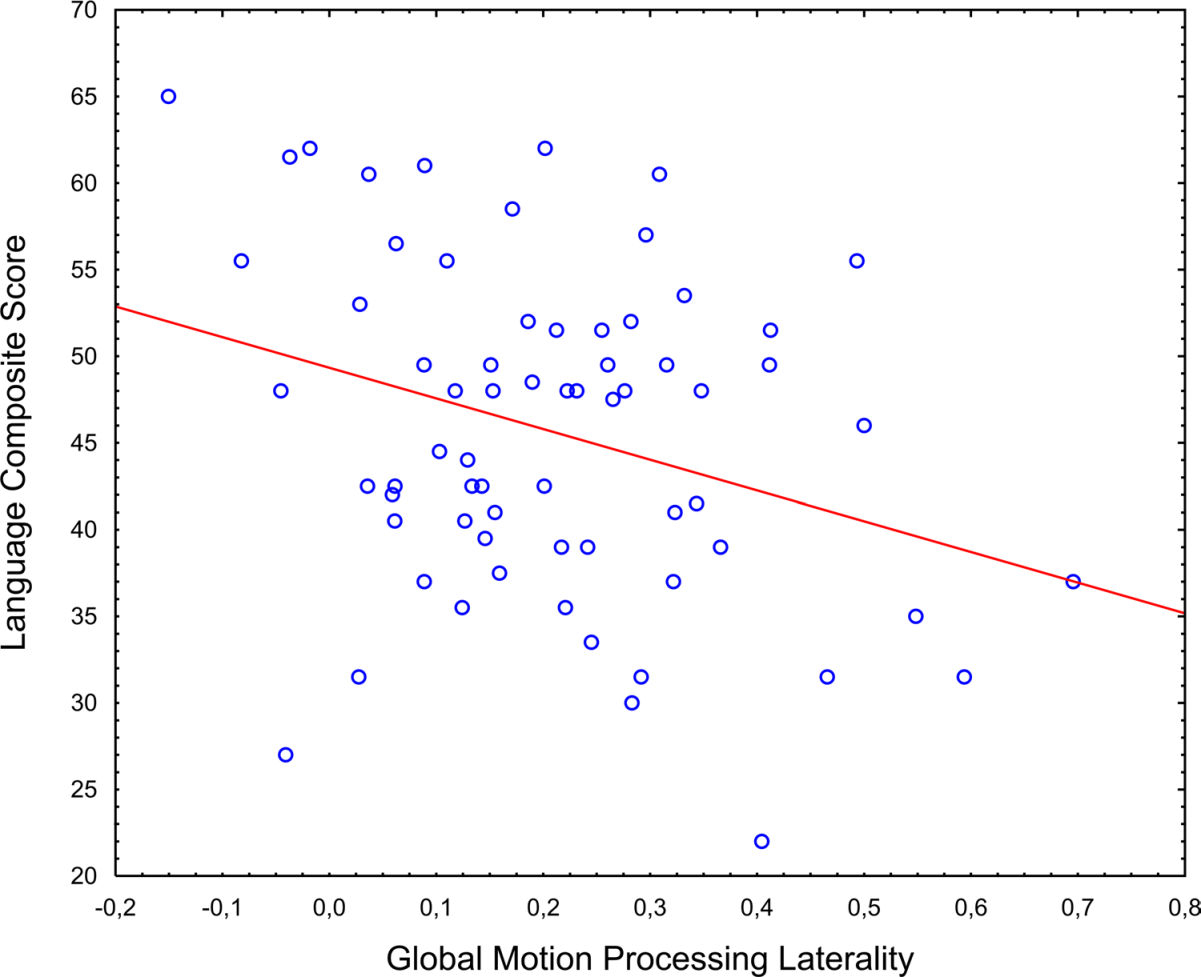
Scatterplot of GMP laterality against MSEL language composite score across all children

The regression analysis with GMP and GFP laterality as independent variables and the mean T-score of the receptive and expressive language subscales as the dependent variable revealed a significant effect of GMP laterality (*F*[1, 64] = 6.51, *p* = 0.013, $\eta_\rho^2$ = 0.092, see Fig. 2) and no effect of GFP laterality (*F*[1,64] = 0.626, *p* = 0.432, $\eta_\rho^2$ = 0.010). The main effect of GMP laterality remained significant when group and sex were added to the model (*F*[1, 56] = 4.20, *p* = 0.045, $\eta_\rho^2$ = 0.070). No other main effects or interactions were significant (all *p*s > . 105).

**Fig. 3:**
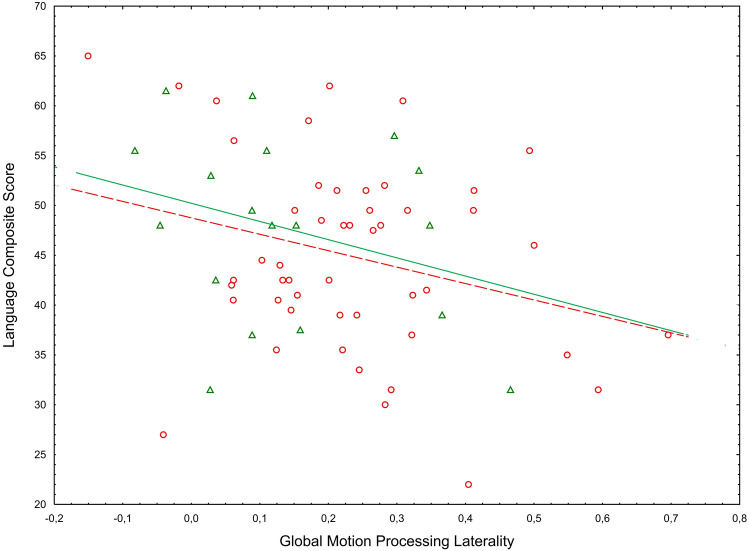
Scatterplot of GMP laterality against MSEL language composite score displayed by group. *Note* Circles represent the children with elevated likelihood of ASD (EL) and triangles represent the children with low likelihood of ASD (LL). The dotted line depicts the regression for the EL group and the solid line depicts the regression line for the LL group. Separate regression lines are provided for descriptive purposes only. Statistically, group did not moderate the association between Global Motion Processing Laterality and the Language Composite Score.

In order to examine the relationship between GMP laterality and language skill in more detail, we performed exploratory correlation analyses between GMP laterality and the receptive and expressive language total scores, separately (Fig. 3). These analyses showed a significant association between GMP laterality and receptive language (*r* = −0.30, *p* = 0.014) whereas the association between GMP laterality and expressive language did not reach statistical significance (*r* = −0.19, *p* = 0.118). The difference between these correlations were, however, not significant (*Z* = −0.66, *p* = 0.509).

### Exploratory Analysis Of The Predictive Relationship Between GMP/GFP and Visual Cognition

**Fig. 4:**
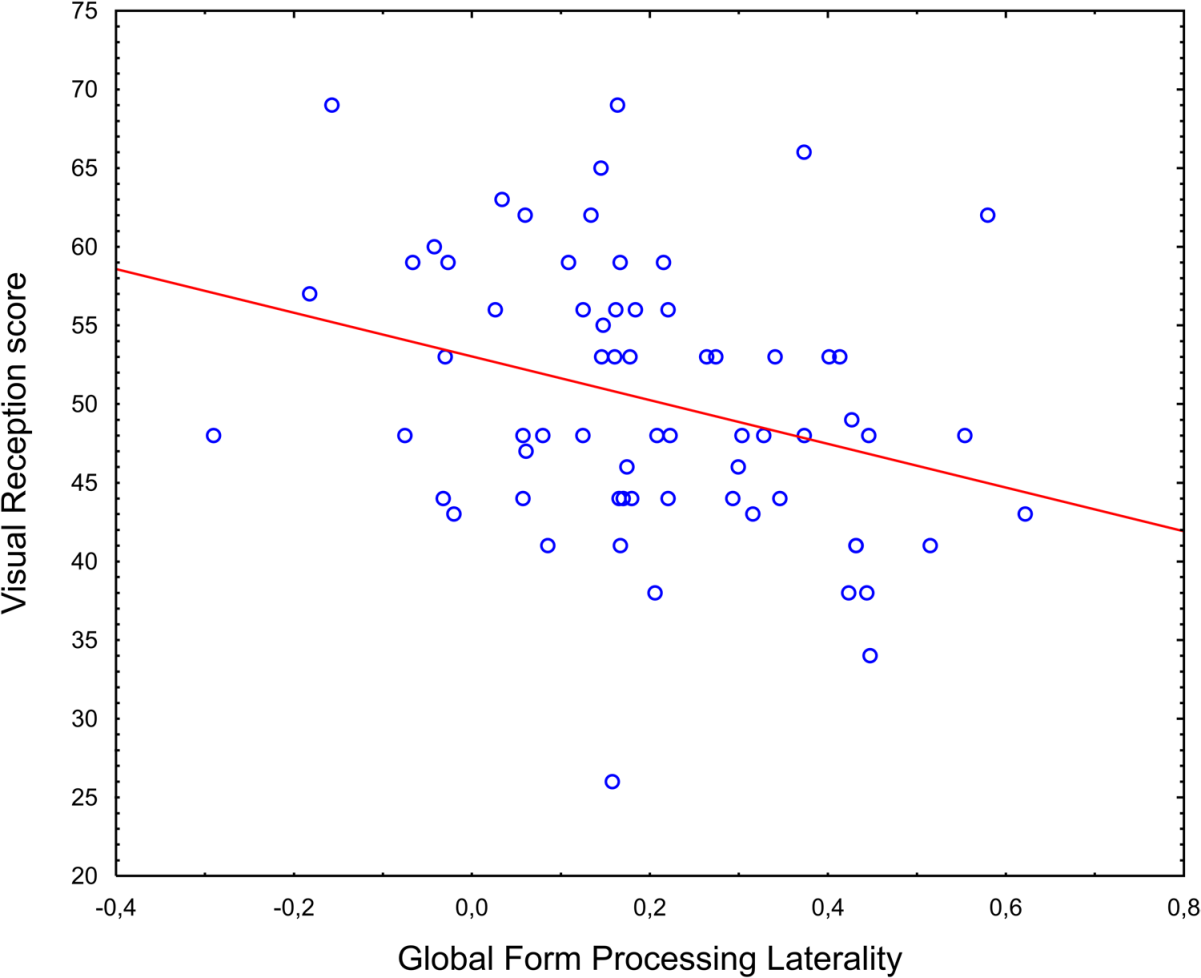
Scatterplot of GFP laterality against MSEL visual reception score across all children

**Fig. 5:**
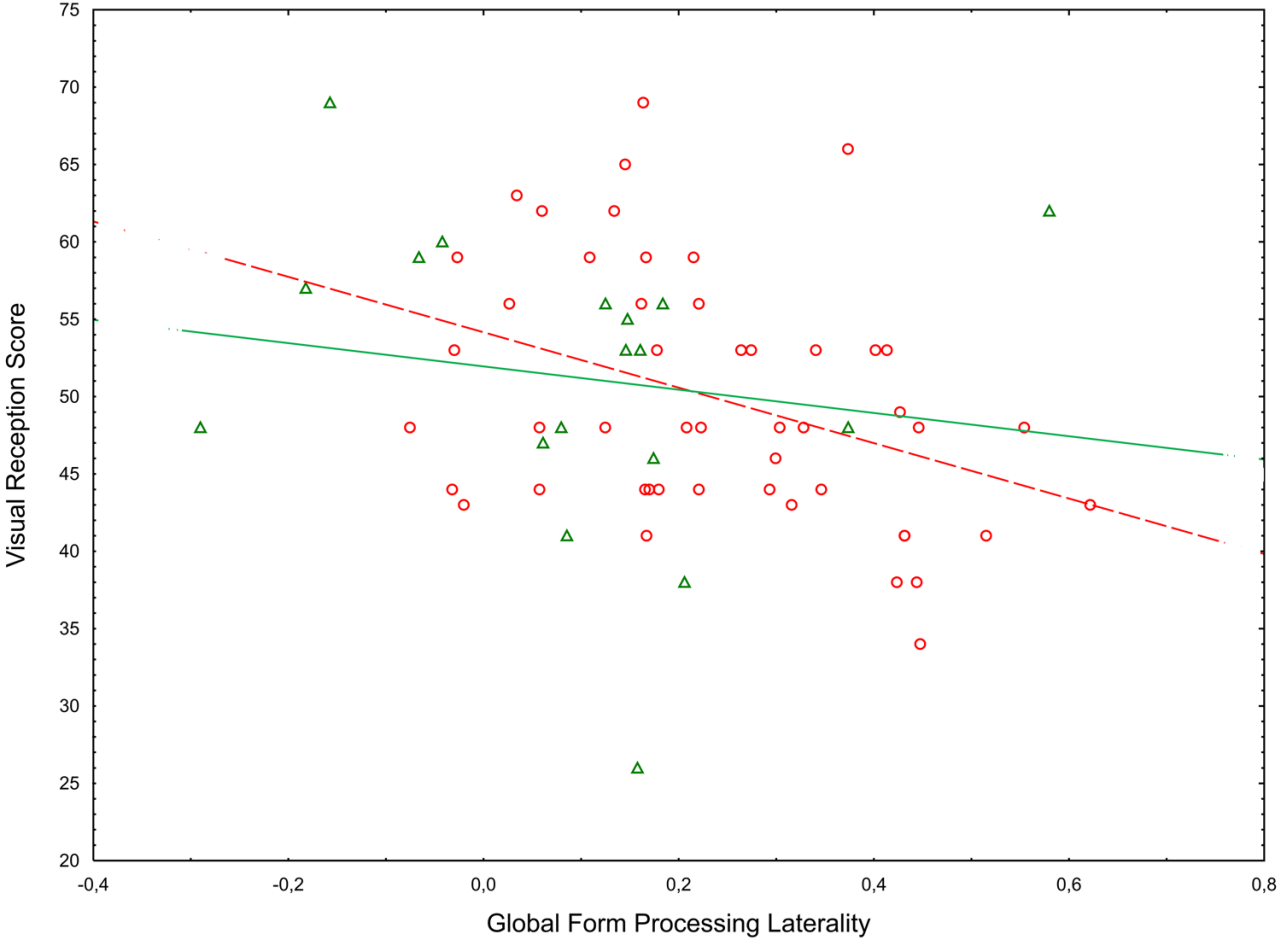
Scatterplot of GFP laterality against MSEL visual reception displayed by groups. *Note* Circles represent the children with elevated likelihood of ASD (EL) and triangles represent the children with low likelihood of ASD (LL). The dotted line depicts the regression for the EL group and the solid line depicts the regression line for the LL group. Separate regression lines are provided for descriptive purposes only. Statistically, group did not moderate the association between Global Form Processing Laterality and Visual Reception Score

The regression analysis with GMP and GFP laterality as independent variables and the visual reception T- score as the dependent variable produced a significant effect of GFP laterality (*F*[1,64] = 6.58, *p* = 0.013, $\eta_\rho^2$ = 0.093, Figs. 4 and 5). The effect of GMP laterality was not significant (*F*[1, 64] = 0.02 *p* = 0.871). The significant association between GFP laterality and visual reception skill held also when group and sex were added to the model (*F*[1, 56] = 8.37, *p* = 0.005, $\eta_\rho^2$ = 0.130). No other significant main effects or interactions were observed (all *p*s = 0.071).

## Discussion

The present study examined the predictive ability of Global Motion Processing (GMP) and Global Form Processing (GFP) at 5 months of age for later motor and language development in children at elevated (EL) and low likelihood (LL) of ASD. It was hypothesized that a higher degree of atypical GMP lateralization at 5 months would be associated with poorer motor as well as language scores at 18 months, whereas no such association would be found for GFP. Results showed early GMP was linked to later language ability, but not motor ability. There was no association between GFP and language or motor ability. In an exploratory arm of the study, a significant association was observed between 5-month GFP and 18-month visual reception. There were no interaction effects with group or sex, suggesting the pattern of results were the same within boys and girls and within EL and LL children. Taken together, results suggest that early GMP is associated with later language ability, but not visual cognition, while GFP is associated with later visual cognition, but not language ability.

The observed association between GMP and language ability is in line with previous research that showed concurrent links between GMP and verbal ability in 2- and 4.5-year-old children (Chakraborty et al., 2017; Thompson et al., 2017). The present findings add to the literature by demonstrating for the first time that early GMP may also be associated with later language ability. There are several possible paths by which GMP atypicalities in infancy could affect language development. Future research examining the potentially mediating role of facial movement processing in the relationship between GMP atypicalities and later language ability is warranted (Shah et al., 2016). For example, evidence suggests that atypical integration of facial movement could affect language development via its impact on socio-cognitive development (Taylor et al., 2020) and/or via visual speech processing (Borowiak et al., 2018). Evidence also exists for additional potential mediators of GMP and later language development, such as attention (Maunsell & Treue, 2006), the integration of information over space and time (Happé & Frith, 2006), signal-from-noise segregation and predictive coding (Van de Cruys et al., 2017).

Notably, early-onset neurodevelopmental alterations are rarely restricted to isolated brain regions and ASD has previously been associated with altered lateralization of various cognitive and motor functions (Paquet et al., 2017). Thus, it appears plausible that the lateralization alterations of GMP observed in the present EL sample are accompanied by additional brain alterations that were not controlled for in the present study. Another possibility may therefore be that the link between atypical GMP laterality and language is driven by co-occurring lateralization alterations in areas more directly related to language. For example, Liu et al. (2019) found evidence for lateralization alterations of the superior longitudinal fasciculus in a group of 6-weeks-old EL infants, compared to LL infants, and these alterations were associated with poorer language outcomes at 18 months of age. More research is needed in order to elucidate the precise mechanisms underlying the observed GMP – language association.

It should be noted that while statistically significant and theoretically interesting, the observed GMP – language association is not strong enough to make individual predictions in a clinically meaningful way. This finding is in line with the multifactorial nature of language development and disorders. On this view, complex traits such as language ability are the sum of a number of underlying capacities, each of which explains only a limited amount of the variance in this ability (Norbury, 2013; Pennington & Bishop, 2009). Nevertheless, it is possible that atypical GMP could be of clinical utility when used as one of several potential indicators of an elevated likelihood of language delays.

Based on previous research linking the dorsal stream to motor-related functions and studies showing concurrent links between GMP and motor functions in children (Chakraborty et al., 2017; Thompson et al., 2017), we had hypothesized that GMP would also be associated with children’s motor development. This prediction was not borne out. One possible explanation for the discrepancy between our findings and those of previous studies may be that whereas the previous studies were cross-sectional, the present study had a longitudinal design and younger participants, at both time points. In addition, there were differences in the specific methods used to assess GMP (i.e., topographical distribution of brain responses, as opposed to functionally determined motor coherence thresholds) making direct comparisons difficult. An interesting target for future research would be to examine to what degree these GMP measures are correlated, and if concurrent associations between topographical brain responses of GMP and motor ability can be found.

The link between GFP and visual cognition observed in the exploratory analysis provides an interesting target for further investigation. The MSEL visual reception sub-domain includes tasks such as discriminating and matching objects by shape (Mullen, 1995), functions that have been shown to be supported by the ventral stream (Goodale & Milner, 1992). As such, findings of the current study are consistent with previous studies of ventral stream function. However, we are not aware of any previous infant research that have examined a possible link between GFP and visual cognition, and the present finding needs to be replicated. Just like the association between GMP and language, the relation between GFP and visual reception did not differ significantly between the EL and LL groups. This suggests that early GFP atypicalities could potentially predict aspects of visuo-cognitive development independent of group status.

The present study has limitations that may be addressed by future studies. First, the sample size was relatively small. In particular, this might have affected the power to detect possible interaction effects with group and sex due to the small sample size of each subgroup (e.g., the LL Female/Male groups consisted of only nine children in each group). Therefore, future studies with larger sample sizes are needed to replicate and validate our findings. In addition, the lack of diagnostic outcome data makes it unclear to what extent our findings may be generalized to children with a clinical diagnosis of ASD. Future research that includes diagnostic outcome and examines potential differences in the GMP-language association between EL and LL children that fulfill ASD diagnostic criteria, and those that do not, are warranted.

## Conclusion

The present infant EEG study suggests that early GMP is associated with later language ability but not visual cognition, while early GFP is associated with later visual cognition but not language ability. We found no indication that these associations were moderated by likelihood of ASD, or biological sex. If confirmed, these new findings could have implications for our understanding of the factors influencing language and cognitive development in ASD. Further research on the potential clinical utility of these findings is warranted.
